# Mental Health Outcomes for Youths With Public Versus Private Health Insurance Attending a Telehealth Intensive Outpatient Program: Quality Improvement Analysis

**DOI:** 10.2196/41721

**Published:** 2022-11-10

**Authors:** Kate Gliske, Katie R Berry, Jaime Ballard, Michelle Evans-Chase, Phyllis L Solomon, Caroline Fenkel

**Affiliations:** 1 Charlie Health Bozeman, MT United States; 2 Center For Applied Research and Educational Improvement University of Minnesota Saint Paul, MN United States; 3 School of Social Policy and Practice University of Pennsylvania Philadelphia, PA United States

**Keywords:** telehealth, telepsychiatry, telemedicine, intensive outpatient, remote outpatient, mental health, quality improvement, routine outcome monitoring, mental health treatment, patient outcome, outpatient program, youth, young adult, depression, suicidal ideation, health outcome, outcome monitoring

## Abstract

**Background:**

COVID-19 exacerbated a growing mental health crisis among youths and young adults, worsened by a lack of existing in-person options for high-acuity care. The emergence and growth of remote intensive outpatient programs (IOPs) is a solution to overcome geographic limitations to care. However, it remains unclear whether remote IOPs engender equivalent clinical outcomes among youths with public insurance (eg, Medicaid) versus private insurance (eg, commercial) given the disparities found in previous research on place-based treatment in both clinical and engagement outcomes.

**Objective:**

This analysis sought to establish, as part of ongoing quality improvement efforts, whether engagement and clinical outcomes among adolescents and young adults attending remote IOP treatment differed between youths with public and those with private insurance. The identification of disparities by payer type was used to inform programmatic decisions within the remote IOP system for which this quality improvement analysis was conducted.

**Methods:**

Pearson chi-square analyses and independent 2-tailed *t* tests were used to establish that the 2 groups defined by insurance type were equivalent on clinical outcomes (depression, suicidal ideation, and nonsuicidal self-injury [NSSI]) at intake and compare changes in clinical outcomes. McNemar chi-square analyses and repeated-measure 2-tailed *t* tests were used to assess changes in clinical outcomes between intake and discharge in the sample overall. In total, 495 clients who attended the remote IOP for youths and young adults in 14 states participated in ≥7 treatment sessions, and completed intake and discharge surveys between July 2021 and April 2022 were included in the analysis.

**Results:**

Overall, the youths and young adults in the remote IOP attended a median of 91% of their scheduled group sessions (mean 85.9%, SD 16.48%) and reported significantly fewer depressive symptoms at discharge (*t*_447_=12.51; *P<*.001). McNemar chi-square tests of change indicated significant reductions from intake to discharge in suicidal ideation (N=470, *χ^2^*_1_=104.4; *P*<.001), with nearly three-quarters of youths who reported active suicidal ideation at intake (200/468, 42.7%) no longer reporting it at discharge (142/200, 71%), and in NSSI (N=430, *χ^2^*_1_=40.7; *P*<.001), with more than half of youths who reported NSSI at intake (205/428, 47.9%) reporting lower self-harm at discharge (119/205, 58%). No significant differences emerged by insurance type in attendance (median public 89%, median private 92%; *P=*.10), length of stay (*t*_416_=−0.35; *P*=.73), or reductions in clinical outcomes (depressive symptom severity: *t*_444_=−0.87 and *P=*.38; active suicidal ideation: N=200, *χ^2^*_1_=0.6 and *P*=.49; NSSI frequency: *t*_426_=−0.98 and *P*=.33).

**Conclusions:**

Our findings suggest that youths and young adults who participated in remote IOP had significant reductions in depression, suicidal ideation, and NSSI. Given access to the same remote high-acuity care, youths and young adults on both public and private insurance engaged in programming at comparable rates and achieved similar improvements in clinical outcomes.

## Introduction

### Background

The US Surgeon General recently declared youth mental health a state of emergency [1]. Nearly half of youths in the United States report feeling persistently sad or hopeless, and 9% report attempting suicide [2]. Left untreated, depression can lead to school dropout, unemployment, substance abuse, violence, and death [3-6]. It is critical for youths with depression to access effective treatments. When depression is severe and suicidality is high, intensive treatment can be life-saving [7]. However, less than half of US counties have facilities with programs for youths with severe mental health needs [8]. Telehealth may help address this critical shortage of services for youths with severe needs as telehealth intensive psychotherapy services can be accessed remotely from any location.

For telehealth to effectively address this gap, it must be able to serve youths of many demographic groups. However, previous research suggests that telehealth in intensive psychiatric services has poorer reach among youths on public insurance than among youths on private insurance [9], and previous research on intensive psychiatric telehealth among adults found poorer outcomes among those who were publicly insured [10]. Most public health insurance for youths is designed for low-income families, “making it imperative to monitor trends in access to services, including appointment attendance, among Medicaid-insured psychiatrically vulnerable youth” [9]. Research on disparities in reach and outcomes by insurance type is critical to ensure that services reach and meet the needs of all youths.

This paper presents the findings from ongoing quality improvement efforts conducted by *Charlie Health*, a national provider of remote intensive outpatient programs (IOPs) for adolescents and young adults whose program staff collect measurement-based clinical outcome data to assess treatment efficacy and meet the quality assurance reporting requirements of payers and providers as part of routine outcome monitoring. Routine outcome monitoring allows for the rapid translation of findings back into the refinement of a clinical care model and can be particularly useful for understanding what is or is not working in a newer treatment modality such as group telehealth [11,12]. The goal of the analysis reported in this paper was to determine (1) whether clinical outcomes improved during treatment and (2) whether programming provided equivalent engagement and clinical outcomes for all clients regardless of payer type. This outcome monitoring is essential to assess whether remote IOPs are engaging and effective for all clients and better understand and inform how the clinical treatment team approaches differences in barriers to care.

### Clinical Outcomes

A primary diagnosis for which many of the youths at Charlie Health seek treatment is major depressive disorder and comorbid behavioral health issues associated with it, such as suicidal ideation and self-harm. As such, 3 of the primary clinical outcomes regularly assessed and analyzed by Charlie Health include depression, suicidal ideation, and nonsuicidal self-injury (NSSI).

Depression is one of the most common mental health disorders among youths and young adults, affecting nearly 1 in 5 [13]. It is linked to a host of difficulties across the life span, including unemployment, substance use disorders, and suicidality [3,4,14,15]. Suicide is the second most common cause of death among youths and young adults [6]. Among high school students, 19% have seriously considered suicide, and 16% have made a suicide plan [16]. Suicidal ideation and attempts are also linked to other high-risk behaviors such as substance and tobacco use [17,18]. NSSI commonly co-occurs with depression among youths and is a significant predictor of future suicide attempts [5]. Some methods of NSSI cause significant physical damage and health risks, and NSSI significantly increases the likelihood of hospitalization among youths with depression and suicidality [19].

### Intensive Outpatient Services

#### Overview

Partial hospitalization, day treatment, and IOPs are important service offerings to address rising mental health needs and prevent emergency room visits and referrals to inpatient care for youths [20]. Moreover, these treatments are crucial service options given that emergency rooms are past capacity and there exists a current shortage of residential services for youths [21]. Furthermore, a growing research base on youth IOPs demonstrates significant symptom reduction and functional improvement [22-24] while being a more cost-effective alternative to inpatient or residential treatment [25,26].

Despite growing evidence of the effectiveness of IOPs in mitigating mental health severity, these programs are not accessible to all youths. Nationally, less than half (45%) of youths with a mental health diagnosis received treatment in the previous year [27] and, in a National Survey of Mental Health Treatment Facilities, only 63% of US counties had a facility providing mental health outpatient treatment for youths and less than half of US counties had a facility with programs for youths with severe mental health needs [8]. Even among young people who find intensive care, long drives and transportation challenges can lead to drop out [28]. Transportation and scheduling barriers are even more common among youths and families with low income [29]. Therefore, telehealth intensive services may address the critical need to tackle barriers such as the lack of local services and transportation challenges.

#### Providing IOPs via Telehealth

##### Overview

For intensive outpatient services to meet the growing mental health needs of youths, they must be both *engaging* and *effective*. Services must be *engaging* to youths and young adults for them to remain in treatment long enough to receive an adequate dose. This requires structuring treatment to address pressing barriers such as transportation and scheduling challenges as well as building strong relationships with both clients and their families. Services must then be *effective* in reducing symptoms and improving functioning.

Telehealth IOPs may address transportation and scheduling barriers that could contribute to engagement, and meta-analyses document that telehealth psychiatric care has equivalent outcomes to those of in-person care [30,31]. However, there is a critical need to investigate potential disparities in engagement and effectiveness by demographic factors [32]. Preliminary research has led to a call for greater attention to treatment disparities by insurance type [9,10,32,33]. Such research is critical for informing equitable care. If there are disparities in engagement, it may be that services have inequitable barriers to participation. If there are disparities in outcomes, there may be differences in either the quality of services by insurance type or in the stressors faced by families by insurance type that require more effective tailoring of services.

##### Telehealth IOPs, Engagement, and Insurance Type

Preliminary research suggests that telehealth attendance among youths with public insurance is lower than among privately insured youths. A study of psychiatric intensive outpatient services that transitioned to telehealth at the start of the COVID-19 pandemic found that adolescents’ attendance was higher only for privately insured youths but not for publicly insured youths [9]. Another investigation of claim data among publicly insured children similarly found that those with the lowest income had overall lower rates of telehealth use [34]. It is notable that research on adults shows no difference in attendance and retention for telehealth between publicly and privately insured individuals, although publicly insured adults had lower attendance for in-person services [10]. This suggests that, for children, attendance to telehealth sessions may be influenced by different barriers from those for adults and that children on publicly funded insurance plans may face other obstacles to attend.

##### Telehealth IOPs, Clinical Outcomes, and Insurance Type

In a recent study of publicly and privately insured adults in remote IOP services, clients with commercial insurance showed significantly greater improvement than those with public insurance despite having comparable treatment engagement [10]. No studies to our knowledge have investigated the differences in outcomes among youths attending telehealth IOP services. However, 3 studies have evaluated differences by insurance type in in-person intensive programs for youths. In each case, youths with private insurance had significantly greater improvements than youths with public insurance. A study of a transdiagnostic child partial hospitalization program found greater reductions in depression and emotional symptoms among children with private insurance relative to those with public insurance [32]. In the only study identified that collected data from multiple reporters, adolescents in an IOP treating self-harm reported no differences by insurance type, but parents of youths with private insurance reported significantly greater behavior improvement than parents of youths with public insurance [28]. A recent study of an IOP for self-harm examined treatment differences by insurance type with the largest sample to date (n=1327 [35]), finding a significantly greater increase in functioning for youths and young adults on private insurance than for those on public insurance. In each of these cases, the authors note that time and the financial commitments of the intensive program may cause greater stressors for youths and families on public insurance than for those on private insurance.

### Aims and Clinical Implications

*Charlie Health* collects clinical outcome data from all clients to track changes in client needs as well as to iteratively inform organization-level processes. The analysis includes comparisons among client subgroups such as those defined by gender, sexual orientation, and—as reported in this paper—insurance type. This approach to quality improvement allows the program to identify the need for differential approaches that could increase engagement and improve patient outcomes. The ultimate goal of all Charlie Health quality improvement efforts is to assess and improve the quality of services for all youths and young adults in the program. Given the existing literature reviewed, the analysis presented in this paper sought to explore (1) whether depression, suicidality, and self-harm improved during treatment and (2) whether exposure to the same program engendered equivalent program engagement and changes in clinical outcomes for all clients regardless of insurance type (public vs private).

## Methods

### Overview

All youths attending Charlie Health are exposed to the same clinical program regardless of insurance type, thus allowing the program to make direct comparisons of engagement and outcomes across the 2 client groups in response to the same treatment. This paper represents one such analysis conducted to better understand the similarities and differences in outcomes and program engagement between clients with 2 different types of insurance: public and private. Data from self-reported intake and discharge assessments along with administrative program engagement tracking metrics (ie, rate of attendance and length of stay [LOS]) were used to explore differences between the 2 insurance subgroups.

### Ethics Approval

This project was reviewed and determined by the University of Pennsylvania Institutional Review Board to qualify as quality improvement, indicating that these activities are not human participant research.

### Client and Program Characteristics

Charlie Health clients come from many geographic regions across the United States, including rural, urban, and suburban communities where there is variable access to mental health services. Relatedly, the program accepts both public and private insurance in 10 of the 12 states in which it operates, removing a common access barrier to services for clients of varying socioeconomic backgrounds. Clients generally present with high-acuity primary and co-occurring mental and behavioral health needs. Approximately half of the clients have recent emergency room visits or inpatient stays related to mental health issues, whereas some clients are initiating mental health treatment for the first time and others are stepping up from a lower level of care. The client population comprises a myriad of marginalized cultural and social identities related to gender and sexual orientation.

Data for clients discharged from treatment between July 21, 2021, and April 28, 2022, were considered for inclusion in the analysis. Client cases were included if they met a minimum engagement threshold defined as ≥7 treatment sessions (approximately 21 hours) and 2 weeks in care and completed both intake and discharge surveys. Engagement criteria were based on neurological evidence suggesting that structural changes occur in response to cognitive and behavioral therapies after approximately 18 hours [36-39]. The resulting 495 cases included all client cases who met these criteria regardless of whether the client completed treatment. Reasons for client discharge before completing treatment vary and include discontinuing treatment against clinician advice, disengagement in treatment sessions, transfer to a higher level of care, and insurance denial.

The Charlie Health IOPs are all telehealth, offering a standard 9 hours of group therapy and an additional 1 hour of individual and 1 hour of family therapy per week. Group sessions are offered during the morning, afternoon, and evening hours to meet the demands of variable work, family, and school schedules. Charlie Health also offers additional optional family programs that include parent support groups that provide optional breakout sessions for parents of clients who are members of the lesbian, gay, bisexual, transgender, and queer community. When clients are admitted to the program, they are placed on a group track that is informed by their mental and behavioral health challenges and demographic characteristics. The program is multimodal in its therapeutic approach, offering evidence-based interventions specific to client needs. For instance, clients with a significant history of trauma are placed in trauma-informed cognitive behavioral therapy groups, and clients who are at high risk of suicide are placed in dialectical behavioral therapy groups. In addition to evidence-based interventions, clients attend general therapeutic processes and experiential groups (ie, mindfulness, creative arts, and music therapy).

### Data Collection Procedures

Clinical outcome data on depression, self-harm, and suicidal risk and behaviors were collected at intake and discharge. Assessments were performed during the clients’ first and last IOP sessions by program staff using a Qualtrics (Qualtrics International Inc) link to the assessment survey. To ensure completeness of the data collected, clients who did not attend their closing IOP group were sent a small financial incentive to complete their discharge survey via an emailed link. All intake and discharge assessment data were downloaded, deidentified, and uploaded to a secure cloud-based folder that was shared with the University of Pennsylvania assessment team, which conducted analyses monthly.

### Measures

Client demographic characteristics were collected at treatment intake (age) and discharge (age, gender identity, and sexual orientation). Treatment episode data came from administrative records that track admission and discharge dates, the number of weeks in treatment, the total number of sessions scheduled and attended, and the discharge reason (eg, treatment completion, leaving against clinical advice, insurance denial, or transferring to a higher or lower level of care).

#### Treatment Engagement

Charlie Health tracks program engagement administratively (vs self-report), monitoring client attendance by session, day, and week as well as overall LOS and type of discharge (ie, treatment completed or discharged for other reasons [insurance denial, referral to a higher level of care, or disengagement from treatment]). The data used to assess treatment engagement differences by insurance type in this analysis included attendance rate measured by the proportion of treatment sessions attended versus those scheduled for the client, LOS measured as the number of weeks between intake and discharge, and type of discharge (treatment complete vs discharged for other reasons).

#### Patient Health Questionnaire Modified for Adolescents

The Patient Health Questionnaire modified for adolescents (PHQ-A) is a screening tool for depression that is administered before and after treatment. The PHQ-A is a 9-item self-report measure that classifies clients into 5 depression severity categories based on their score from 0 to 27: minimal (0-4), mild (5-9), moderate (10-14), moderately severe (15-19), and severe (20-27) [40]. The instructions ask clients to rate how bothered they have been by symptoms of depression over the past 2 weeks (ie, *Feeling down, depressed, irritable, or hopeless* or having *Thoughts that you would be better off dead, or of hurting yourself in some way*). Responses are rated on a 4-point Likert scale from 0 (not at all) to 3 (nearly every day) wherein the sum scores range from 0 to 27. The PHQ-A has been established as an excellent diagnostic screening tool for depression in adolescent samples [41,42]. Reliability in this sample was excellent at admission and discharge, with a Cronbach α of .91 at both time points.

#### Suicide Risk

Suicidality was measured by the program using the 5-item Ask Suicide-Screening Questions (ASQ) Toolkit. The ASQ was developed as a suicidal risk screening toolkit for health care providers wherein, if clients responded *yes* to any of items 1 through 4, they would be screened as *positive* for suicide risk and asked an additional question about current suicidal ideation. The ASQ 1 and ASQ 2 ask clients to reflect on passive suicidal ideation (*thinking you would be better off dead* and *felt that you or your family would be better off if you were dead),* the ASQ 3 asks about active ideation (*In the past week, have you had thoughts of killing yourself*), and the ASQ 4 asks about previous attempts (*Have you ever tried to kill yourself?).* The 4 items were validated in a pediatric sample (ages 10-21 years) wherein the measure correctly identified 96.9% of the sample that screened *positive* for suicide risk [43].

#### NSSI Measure

NSSI was assessed by the program using Criterion A of the Alexian Brothers Assessment of Self-Injury (ABASI) scale [44]. The ABASI was created to measure clinical severity of NSSI behaviors. The scale is broken up into 4 criteria that mirror the Diagnostic and Statistical Manual of Mental Disorders criteria for NSSI disorders. For this assessment, the 21-item Criterion A subscale was used to assess self-harm frequency and clinical severity. The subscale asks clients to report the number of days they engaged in any one of 21 different types of self-harm (ie, *Cut yourself enough to tear the skin and/or bleed* or *scratched, rubbed, or pinched at your skin to the point of bruising or bleeding*) over the previous 30 days. Washburn et al [44] suggest a cutoff score of 5 total days for any one NSSI type to be considered clinically significant NSSI.

### Data Preparation

Before the analysis, 8 new variables were created, equivalence between insurance groups was assessed, and tests of normality of continuous variables were conducted.

#### Newly Created Variables of Outcome Change and Engagement

##### Depression

In total, 2 change variables were computed using the continuous PHQ-A scores. The first change variable was created by subtracting the raw PHQ-A intake score from the raw score at discharge, resulting in a continuous change score. To create the second change variable, continuous PHQ-A scores were recoded to classify clients into symptom severity categories based on their scores at intake and discharge, with scores of 0 to 4 indicating 1 (minimal), scores of 5 to 9 indicating 2 (mild), scores of 10 to 14 indicating 3 (moderate), scores of 15 to 19 indicating 4 (moderately severe), and scores of 20 to 27 indicating 5 (severe). Next, a change variable representing whether symptom severity improved between intake and discharge was created by subtracting the discharge severity classification variable from the intake severity classification variable and recoded with 1=*improved* (decreased at least one severity classification), 0=*not improved* (stayed the same or increased in severity classification), and −1=*worsened* (increased 1 severity class). Finally, to assess for changes in depression severity classification, the change variable was recategorized to classify clients as 1=*improved* (dropped 1 severity classification) or 0=*not improved* (stayed the same or worsened in severity class).

##### Suicide Risk

For this evaluation, 2 change variables were computed to assess outcomes by group. The first was a screening status change variable that categorized clients based on improvement wherein clients were classified as *improved* if their screening status changed from positive to negative across time and classified as *not improved* if their status remained positive or transitioned from negative to positive. First, a composite score was created for intake and discharge ASQ items 1 to 4. Then, 2 screening status variables were created wherein clients were categorized as 1=*positive* if their score was >0 and 0=*negative* if their composite score was equal to 0. Next, a change variable was created by subtracting the discharge screening variable from the intake screening variable. This variable was then recoded to reflect changes in screening status, which classified clients as 1=*improved* if their screening status change variable was equal to 1 and 0=*not improved* if their change score was either 0 (indicating no change) or −1 (indicating a negative prescreen status and positive postscreen status).

To assess for changes in active suicidal ideation, a change variable for the ASQ 3 was computed by subtracting the discharge assessment ASQ 3 score (1=*yes* and 0=*no*) from the intake response score to categorize clients as 1=*improved* (“yes” at intake and “no” at discharge) or 0=*not improved* (“yes” at intake and discharge or “no” at intake and “yes” at discharge).

##### NSSI Variable

In total, 2 change variables were created to explore differences in change in NSSI. First, scores on each of the 21 types of NSSI were recoded into dichotomous variables where 1=*met the criteria* (score of ≥5 days) and 0=*did not meet the criteria* (score of <5 days). A composite score for the 21 dichotomous items was used to create criteria variables for intake and discharge where 0=*did not meet the criteria* and 1=*met the criteria* (a score of >1 would indicate that the client met the criteria for at least one subtype). To assess change, a difference variable was created by subtracting the discharge criteria variable from the intake criteria variable. Finally, this variable was transformed into a dichotomous variable where 1=*improved* (difference score of 1) or 0=*not improved* (difference score of 0 or −1).

The second change variable created reflects a total change in frequency across subtypes of NSSI. To calculate this variable, 2 sum scores were computed across subtypes of NSSI at intake and discharge. The change variable was computed by subtracting the discharge frequency score from the intake frequency score. The instructions asked clients to reflect on their frequency of self-harm over the past month; therefore, a cap of 30 was used on total scores to improve the interpretability of the total score.

##### Program Engagement

To explore differences in engagement by group, 2 variables were created that reflected attendance rate and LOS. The attendance rate variable was created by dividing the total number of sessions attended by the client by the total number of sessions scheduled. The LOS variable was computed by subtracting the clients’ discharge date from their admission date and dividing by 7 to calculate the total number of weeks attended by the client.

#### Missing Data

The electronic survey used to collect client responses does not use a force response mechanism; as such, the sample sizes for each of the resultant analyses may differ slightly. The number of clients who met the eligibility criteria was 495; however, given the authors’ decision not to use imputation, the range of sample sizes in the subsequent analyses ranged from 459 to 495 (data coverage of ≥92.7% for each analysis).

#### Test of Normality

To inform decisions about the use of parametric versus nonparametric tests, the distributions for all continuous variables included in this analysis were evaluated for normality. The PHQ-A, NSSI frequency scores, and LOS were normally distributed; however, both age and attendance rate were positively skewed. Therefore, nonparametric tests were determined to be most appropriate for the analysis of the latter 2 variables.

#### Equivalence of Insurance Groups at Intake

A series of tests was conducted to assess the equivalence of the 2 insurance groups (public insurance vs private insurance) at intake that could plausibly explain differences at discharge. Independent *t* tests (2-tailed) were conducted using the PHQ-A intake scores and NSSI frequency. Differences at intake in depression symptom severity classification were also tested using the chi-square test of independence.

### Data Analysis Strategy

Descriptive statistics were used to describe the sample as a whole by demographic and baseline clinical characteristics. Demographic characteristics included age and self-identified gender and sexual orientation. Clinical characteristics comprised baseline clinical assessment scores on the PHQ-A, ABASI, and ASQ.

### Program Engagement

Program engagement was calculated for the sample as a whole. Differences between insurance groups were analyzed using a Mann-Whitney *U* test of median differences for attendance, an independent-sample *t* test (2-tailed) of mean differences for LOS, and chi-square analysis for discharge type.

### Program Effectiveness

Before testing group differences in clinical outcomes by insurance type, changes in outcomes between intake and discharge across all clients were tested for each outcome variable. Differences by insurance type were then tested for those outcomes and were found to show significant improvement between time points.

#### Depression

A repeated-measure 2-tailed *t* test was run on the sample as a whole, and an independent-sample *t* test (2-tailed) was run on the continuous PHQ-A change variable. A McNemar chi-square test was used to assess the number of clients who moved from the moderate to severe depression classification at intake to a lower severity class at discharge. Two chi-square analyses were run on the dichotomous PHQ-A improvement variable: a McNemar chi-square test for the sample as a whole and a Pearson chi-square test to compare the 2 groups.

#### Suicide Risk and Ideation

McNemar chi-square analyses were run to explore changes in suicide risk and active suicidal ideation across groups in a subsample of youths who screened positive at intake. A Pearson chi-square test of independence was run to compare group differences within the subsample.

#### NSSI Variable

To test changes in the sample as a whole, a McNemar chi-square test was used between intake and discharge on the screening variable, and a repeated-measure 2-tailed *t* test was run for NSSI frequency. To explore between-group differences, a Pearson chi-square analysis was run on the NSSI improvement variable, and an independent-sample *t* test (2-tailed) was used to test changes in NSSI frequency. The chi-square analyses of between-group differences in NSSI were run on a subsample of clients who met the criteria at intake (205/459, 44.6%), excluding those who did not meet the criteria at intake.

## Results

### Equivalence of Insurance Groups at Intake

Differences in PHQ-A scores were nonsignificant between the public (mean 13.28, SD 7.58) and private (mean 14.35, SD 7.95) insurance groups (t_475_=−1.297; *P*=.20). Similarly, no significant differences were found in NSSI frequency between the public (mean 12.40, SD 12.40) and private (mean 11.77, SD 11.77) insurance groups (t_455_=0.49; *P*=.63). The results of a chi-square test on significant differences indicated no significant difference in severity classification by insurance type (N=477, *χ*^2^_1_=6.3; *P=*.18). The results of a chi-square analysis conducted with suicide risk status also indicated no significant differences between the groups at intake (N=491, *χ*^2^_1_=0.0; *P=*.98). Similarly, no significant differences were found on the ASQ 3 (N=490, *χ*^2^_1_=3.2; *P=*.07). Finally, differences in NSSI clinical severity status were tested using chi-square analysis. The findings indicated no difference between the groups concerning the proportion of clients who met the criteria versus those who did not (N=459, *χ*^2^_1_=0.1; *P=*.74). Given that no significant differences were found at admission on clinical characteristics, the 2 insurance groups were assumed to be equivalent; consequently, these scores were not included as covariates in the main analyses. See Table 1 for additional details.

**Table 1:** Chi-square clinical differences at intake.

				Insurance group					Chi-square (*df*)			*P* value
				Public		Private						
**Depression severity**									6.3 (4)^a^			.18
	**Minimal**											
		Participants, n (%)	19^b^ (15.8)		52^c^ (14.6)							
		Standardized residual	0.30		−0.20							
	**Mild**											
		Participants, n (%)	26^b^ (21.7)		55^c^ (15.4)							
		Standardized residual	1.20		−0.70							
	**Moderate**											
		Participants, n (%)	20^b^ (16.7)		67^c^ (18.8)							
		Standardized residual	−0.40		0.20							
	**Moderately severe**											
		Participants, n (%)	26^b^ (21.7)		61^c^ (17.1)							
		Standardized residual	0.90		−0.50							
	**Severe**											
		Participants, n (%)	29^b^ (24.2)		122^c^ (34.2)							
		Standardized residual	−1.50		0.80							
**Suicide risk status**									0.001 (1)^d^			.98
	**Positive**											
		Participants, n (%)	88^e^ (71)		260^f^ (70.8)							
		Standardized residual	0.00		0.00							
	**Negative**											
		Participants, n (%)	36^e^ (29)		107^f^ (29.2)							
		Standardized residual	0.00		0.00							
**ASQ 3^g^**									3.23 (1)^h^			.07
	**No**											
		Participants, n (%)	62^i^ (50.4)		219^f^ (59.7)							
		Standardized residual	−1.00		0.60							
	**Yes**											
		Participants, n (%)	61^i^ (49.6)		148^f^ (40.3)							
		Standardized residual	1.20		−0.70							
**NSSI^j^ criteria status**									0.11 (1)^k^			.74
	**Met criteria**											
		Participants, n (%)	59^l^ (48.4)		157^m^ (46.6)							
		Standardized residual	0.20		−0.10							
	**Did not meet criteria**											
		Participants, n (%)	63^l^ (51.6)		180^m^ (53.4)							
		Standardized residual	−0.20		0.10							

^a^N=477.

^b^N=120.

^c^N=357.

^d^N=491.

^e^N=124.

^f^N=367.

^g^ASQ 3: Ask Suicide-Screening Questions 3.

^h^N=490.

^i^N=123.

^j^NSSI: nonsuicidal self-injury.

^k^N=459.

^l^N=122.

^m^N=337.

### Client Demographic Characteristics

There were a total of 1461 clients admitted to the program during the data collection period, of whom 995 (68.1%) met the engagement criteria of attending ≥7 sessions. Of those who did not meet the engagement criteria, 62.9% (293/466) were discharged within the first week of treatment at Charlie Health (≤3 sessions). Of the 995 remaining clients, 500 (50.3%) did not complete a discharge survey, resulting in 495 client cases included in the analysis.

The average age of the clients was 16.7 years, and over three-quarters of the sample were adolescents (385/495, 77.8%). The age range of this sample was 11 to 35 years (wherein clients aged between 11 and 25 years comprised 477/495, 96.4% of the sample; the remaining were between the ages of 26 and 35 years). Over half of the clients identified as women (249/495, 50.3%), almost a quarter (113/495, 22.8%) self-identified as a nonbinary, and 20.1% (99/492) self-identified as transgender. Most clients (321/495, 64.8%) identified as members of the lesbian, gay, bisexual, transgender, and queer community, with 34.9% (173/495) identifying as heterosexual. Approximately half of the clients (248/494, 50.2%) reported an admission to a higher level of care or a visit to an emergency room in the 30 days before IOP admission. The only significant difference in demographic factors between groups was transgender identity such that a significantly greater proportion of clients with public insurance identified as transgender compared with clients with private insurance (183/495, 37% vs 86/495, 17.4%). The ratio of public to private insurance among Charlie Health clients (370/495, 74.7% have private insurance and 125/495, 25.3% have public insurance) mirrors that of the national population aged <65 years (67.7% have private insurance and 20.7% have public insurance) [45].

### Program Engagement

Table 2 presents the results of the Mann-Whitney *U* test and independent-sample 2-tailed *t* tests that were run to determine if there were differences in the attendance rate and LOS between insurance types. The median attendance of the sample as a whole was 91% (mean 85.9%, SD 16.48%). The results of the Mann-Whitney *U* test were nonsignificant, indicating no significant difference in attendance rate between clients with public (123/491, 25.1%; median 89%) and private (368/491, 74.9%; median 92%) insurance types (*U=*24,858.5; *z* score 1.65; *P=*.10).

The average LOS for the sample as a whole (N=491) was 10.8 weeks (SD 4.60). The results of the independent-sample *t* test (2-tailed) comparing LOS across insurance groups indicated no statistically significant difference between clients with public (123/491, 25.1%; mean 10.54) and private (368/491, 74.9%; mean 10.88) insurance (t_489_=−0.72; *P=*.47).

Finally, significant differences were assessed on discharge reason by insurance type. The results of the chi-square analysis indicated no significant differences between the groups, wherein 67.2% (84/125) of clients with Medicaid insurance completed treatment and 71.2% (259/364) of clients with commercial insurance completed treatment (N=489, *χ*^2^_1_=0.7; *P=*.43).

**Table 2:** Client length of stay (LOS) and attendance by insurance type.

			Values		Mean difference
**LOS** **,** **mean (SD)**					
	Public	10.54 (4.79)		−0.34	
	Private	10.88 (4.54)		N/A^a^	
**Attendance, median; mean rank^b^**					
	Public	89%; 227.90		N/A	
	Private	86.55%; 252.02		N/A	

^a^N/A: not applicable.

^b^*P*=.10 (2-tailed).

### Depression

The repeated-measure 2-tailed *t* test indicated that, across the sample as a whole, clients scored significantly lower on the PHQ-A at discharge compared with at intake (t_447_=12.51; *P<*.001). The independent-sample *t* test (2-tailed) comparing changes in PHQ-A score by insurance type found no significant difference between the groups (t_444_=−0.87; *P=*.38), suggesting that clients improved significantly from intake to discharge in depression severity regardless of insurance type. The McNemar chi-square test assessing change in symptom severity among the whole sample between intake and discharge indicated that a significant number (163/192, 84.9%) of those who scored moderate to severe at intake (N=650, *χ*^2^_1_=17.6; *P<*.001) scored in a lower category at discharge, 83.2% (99/119) of clients who were classified as “moderately severe” at intake moved down at least one classification at discharge (N=434, *χ*^2^_1_=12.3; *P<*.001), and 73.2% (82/112) of clients who were classified as “moderate” at intake dropped at least one classification level by discharge (N=296, *χ*^2^_1_=9.7; *P<*.001). Pearson chi-square tests comparing changes in symptom severity between the 2 insurance groups indicated no significant differences (N=446, *χ*^2^_1_=0.1; *P*=.71). See Table 3 for more details.

**Table 3:** Chi-square analysis of difference in depression severity by insurance type (N=446).

				Insurance group					Total			Chi-square (*df*)				*P* value	
				Public (n=111)		Private (n=335)											
**Improved between intake and discharge**													0.1 (1)				.71
	**Not improved**																
		Participants, n (%)	47 (42.3)		135 (40.3)		182 (40.8)										
		Standardized residual	0.30		−0.10		N/A^a^										
	**Improved**																
		Participants, n (%)	64 (57.7)		200 (59.7)		264 (59.2)										
		Standardized residual	−0.20		0.10		N/A										

^a^N/A: not applicable.

### Suicide Risk

The McNemar chi-square test assessing the number of clients who improved in symptom severity between intake and discharge across the sample as a whole found that, of the clients who screened positive at intake (330/470, 70.2%), significantly fewer (115/330, 34.8%) screened positive at discharge (*P<*.001). The results of the Pearson chi-square analysis indicated no significant differences in the number of clients who improved between intake and discharge by insurance type (N=330, *χ*^2^_1_=0.1; *P*=.89). See Table 4 for more details.

The McNemar chi-square test assessing the number of clients who improved in active suicidal ideation symptoms between intake and discharge across the sample as a whole found that, of the clients who screened positive at intake (201/470, 42.8%), significantly fewer (92/201, 45.8%) reported active suicidal ideation at discharge (N=470, *χ*^2^_1_=19.2; *P<*.001). The results of the Pearson chi-square analysis indicated no differences in the number of clients who improved between intake and discharge between the 2 insurance types (N=200, *χ*^2^_1_=0.6; *P*=.49). In other words, both groups improved similarly on active suicidal ideation from intake to discharge (see Table 4 for additional details).

**Table 4:** Chi-square analysis of differences in suicide risk by insurance type.

Screening status between intake and discharge					Insurance group							Total				Chi-square (*df*)				*P* value
					Public			Private												
**Suicidal ideation**																0.06 (1)^a^				.89
	**Not improved**																			
		Participants, n (%)	28 (33.7)			87 (35.2)			115 (34.8)											
		Standardized residual	−0.20			0.10			N/A^b^											
	**Improved**																			
		Participants, n (%)	55 (66.3)			160 (64.8)			215 (65.2)											
		Standardized residual	0.10			−0.10			N/A											
	Total, n (%)			83 (100)			247 (100)			330 (100)										
**Active suicidal ideation**																0.56 (1)^c^				.49
	**Not improved**																			
		Participants, n (%)	19 (32.8)			39 (27.5)			58 (29)											
		Standardized residual	0.50			−0.30			N/A											
	**Improved**																			
		Participants, n (%)	39 (67.2)			103 (72.5)			142 (71)											
		Standardized residual	−0.30			0.20			N/A											
	Total, n (%)			58 (100)			142 (100)			200 (100)										

^a^N=330.

^b^N/A: not applicable.

^c^N=200.

### NSSI Variable

The repeated-measure 2-tailed *t* test run on all clients (n*=*430) indicated a significant reduction in NSSI frequency from intake (mean 12.09, SD 12.34) to discharge (mean 6.08, SD 9.38; t_429_=10.41; *P<*.001) across the 2 groups. The independent-sample *t* test (2-tailed) evaluating differences between insurance groups in changes in frequency (total days) of NSSI was not significant (t_426_=−0.98; *P*=.33). Thus, clients appear to have improved in NSI frequency across the sample regardless of insurance type.

The McNemar chi-square test comparing changes in symptom severity among those clients who met NSSI criteria at intake (205/428, 47.9%) indicated a significant change such that 58% (119/205) did not meet the criteria at discharge (N=430, *χ*^2^_1_=40.7; *P*<.001). The results of the Pearson chi-square analysis indicated no significant difference between the public and private health insurance groups in the changes experienced between intake and discharge in NSSI symptom severity (N=205, *χ*^2^_1_=0.9; *P*=.35). See Table 5 for additional details.

**Table 5:** Chi-square analysis of differences in nonsuicidal self-injury by insurance type (N=205).

				Insurance group				Total		Chi-square (*df*)			*P* value	
				Public (n=55)		Private (n=150)								
**Improved between intake and discharge**											0.9 (1)			.35
	**Not improved**													
		Participants, n (%)	26 (47.3)		60 (40)		86 (42)							
		Standardized residual	0.60		−0.40		N/A^a^							
	**Improved**													
		Participants, n (%)	29 (52.7)		90 (60)		119 (58)							
		Standardized residual	−0.50		0.30		N/A							

^a^N/A: not applicable.

## Discussion

### Principal Findings

The aims of the quality improvement analysis reported in this paper were to (1) assess whether clinical outcomes improved during treatment and (2) assess for differences in engagement or outcomes between adolescents and young adults engaging in remote IOPs on either public or private insurance. The findings of this analysis support the effectiveness of remote IOP treatment in reducing clinical symptoms across all clients, including reduced depression, suicidality, and self-harm. The findings indicate reduced symptoms regardless of insurance type; no differences among youth clients by private or public insurance type emerged across the clinical outcomes tested, evincing similar reductions in depressive symptoms, suicidal ideation, and NSSI. Similarly, youths on public and private insurance were equivalently engaged in treatment, attending treatment for comparable lengths of time and both groups attending nearly all scheduled group sessions (median 91%; mean 85.9%, SD 16.48%) during their treatment stay.

Across the entire sample and regardless of insurance type, youths in remote IOP reported significantly fewer depression symptoms at discharge, with nearly 60% (264/446, 59.2%) of patients evidencing a clinically significant reduction. Of the youths who were actively suicidal at intake, 71% (142/200) no longer reported the same at discharge. Finally, more than half (119/205, 58%) of those who met the criteria for clinical NSSI at intake no longer met the criteria for NSSI at discharge, indicating a significant decline in self-harming behavior. These results provide preliminary support for both the effectiveness of remote IOPs for adolescents and youths with complex mental health needs and the comparable effectiveness of group telehealth among publicly and privately insured patients.

These findings contrast with previous research comparing telehealth IOP outcomes by insurance type that found disparities in outcomes for adults [10] as well as disparities in in-person IOP outcomes for children [32], youths [28], and young adults [35]. In studies addressing youths and young adults, these different findings may be explained by the handling of dropout as these studies appear to have included all clients who initiated treatment [28,35]. It may be that there are higher dropout levels among clients on public insurance and that the resulting effect on outcomes from a smaller dose of treatment is inadequate for symptom improvement. However, a study of a child partial hospitalization program used a nearly identical methodology including only those who stayed for 2 weeks and completed both intake and discharge assessments, finding greater reductions in depression and emotional symptoms for youths on public insurance [32]. As their study focused on children aged 7 to 13 years, it may be that there are differences in treatment outcomes and disparities between children and young adolescents and the adolescents and young adults in our sample. This study also found that families with greater recent stressors had smaller treatment gains even after controlling for insurance type. The authors suggest that families with state-funded insurance may have had “additional stressors that created barriers to treatment use, such as difficulties with transportation and inadequate social supports” [32]. It may be that the telehealth option of these services posed lower stressors on the family, leading to more equitable outcomes.

This quality improvement analysis also found that program attendance among clients who met the inclusion criteria (at least 2 weeks of programming and 7 sessions attended) did not significantly differ in their engagement, both in the total number of weeks and rate of attendance to sessions. This finding also runs counter to recent research that found that, when adolescents were transitioned from in-person to telehealth IOP services during the COVID-19 pandemic, publicly insured and lower-income clients had significantly lower attendance rates [9]. The differences in the findings may be due to the different handling of dropout. In their study, Childs et al [9] measured attendance to all scheduled appointments. It may be that families with public insurance have greater initial barriers and higher early dropout rates. The comparable attendance and outcomes among those who engage suggests that telehealth IOP services can be accessible and effective across insurance types.

Previous studies have identified childcare, transportation, and scheduling challenges as primary barriers to attendance for low-income families [46,47]. Telehealth obviates some of these structural barriers to accessing intensive mental health treatment. Evening and weekend hours can further address barriers to attendance among low-income families [48] and particularly among families with public insurance. Children with public insurance are less likely to have a usual source of care during nighttime or weekend hours and are more likely to experience a delay in receiving care because they could not go when services were open or because of transportation challenges; these differences are significant even after controlling for health, demographic, and socioeconomic differences [49]. Charlie Health has carefully designed its scheduling for accessibility to all clients; services are offered during morning, afternoon, and evening hours to match demanding schedules. The next step in this quality improvement analysis would be to explore the outliers—those adolescents and young adults who had lower than average engagement and clinical improvement—to better understand barriers that might affect this subpopulation of clients who are within the reach of influence of Charlie Health.

Recent systemic changes likely influenced the findings of this analysis in the desired direction. For example, as a result of the pandemic, access to technology and the internet increased in many areas, which, in turn, has facilitated access to telehealth care regardless of income or resources [50]. Ultimately, the analyses conducted in this study suggest that, given access to the same remote high-acuity care, youths and young adults engaged in programming at comparable rates and achieved similar improvements in clinical outcomes regardless of insurance type.

### Strengths and Limitations

These findings should be interpreted within the limitations of the available data, the most notable of which is that the inclusion criteria restricted cases to only those of clients who met the engagement threshold. This likely introduced a selection bias wherein exploring differences in early disengagement may have revealed significant differences between insurance groups. In this project, demographic data were not collected at intake, so it was not possible to assess differences in dropout by demographics. However, the results of this assessment were meant to inform and assess the program from which the client cases came, which is why the survey collection criteria predicated participation in at least 2 weeks of programming to be eligible for discharge surveys. However, future quality improvement efforts should be made to explore potential differences in early engagement that may necessitate program improvement. Future work should also assess a larger range of demographic factors. Notably, rural populations have less access to the internet than urban populations, although the divide is narrowing [51]. Studies are needed to assess the role of geographic location in telehealth IOP engagement.

There are also inherent limitations to solely relying on client self-report data in assessing meaningful clinical change. The data used in the pre-post analyses in this study were collected at 2 distinct points: intake and discharge. Thus, there is no way of knowing if there were specific facilitators or barriers to engagement and treatment effectiveness that emerged throughout the course of treatment. Furthermore, the timing of survey dissemination may have influenced clients’ responses in either direction—for instance, it has been noted by clinical staff that clients report a range of intense positive and negative emotions at the time of intake and discharge. Thus, responses about clinical symptoms may be influenced by heightened emotions elicited at these 2 points (minimizing or exaggerating clinical severity). However, for the purposes of this preliminary quality improvement analysis, the available data proved sufficient to explore high-level differences in engagement and outcomes by insurance type. Future analyses would be strengthened by the inclusion of observational data provided by program staff or treatment-involved family members. Additional data points throughout the course of treatment may also provide a more balanced and nuanced narrative of treatment experiences beyond what can be inferred from the pre- and postsurvey data.

A notable strength of the quality improvement analysis conducted in this study was the ability to compare outcomes and engagement between clients using public and private insurance who participated in the same program at the same time. Research has long noted that publicly insured clients have significantly fewer treatment options compared with privately insured clients [52,53]. Thus, comparing outcomes between populations with private and public health insurance precludes the investigation of the moderating influence of variable program quality as publicly insured clients have fewer options and likely fewer quality options. Poorer comparative outcomes that disfavor publicly insured clients may consequently affirm the damaging stereotypes that this population is less capable of clinical improvement [46]. However, these analyses remove the variable influence of program quality and suggest that socioeconomic factors are not deterministic of treatment outcomes, further impressing the importance of providing equitable access to quality treatment regardless of insurance type.

### Implications

The finding that youths improved regardless of insurance type has direct implications for practice. Previous research suggests that low socioeconomic status adversely affects the likelihood of youths benefiting from mental health treatment [46]. However, the findings of this assessment demonstrate equitable outcomes among youths of varying socioeconomic status using health insurance as a proxy. A notable strength of the program setting is the variable times offered for groups, which addresses a barrier to services frequently shared by families with lower incomes or on public insurance [48,49]. Furthermore, the provision of services on a web-based platform may remove some of the common barriers to service attendance that caregivers report related to transportation, childcare, and time off work [47]. Mental health intensive outpatient service providers considering expansion to publicly insured clients should consider variable times for service offers and remote alternatives.

### Conclusions

Given that these analyses investigated services from a multistate psychiatric care provider for youths and young adults, this assessment is larger in scope than previous investigations of outcomes by insurance type among telehealth intensive psychiatric services. This study contributes to the currently limited evidence base on disparities by insurance type for telehealth intensive psychiatric services. The results suggest that adolescents and young adults on public and private insurance engage in remote IOPs at similar rates, achieving comparable improvements in depressive symptoms, NSI, and suicidal ideation. This suggests that, when given access to the same quality of intensive care in a remote, flexible scheduling format, youths and young adults on either public or private insurance have equal engagement and outcomes. Remote IOPs lead to reduced symptoms for youths with mental health needs across insurance types at a time when such services are needed by millions of adolescents and young adults who do not frequently have access to care because of geographic or financial limitations.

## Abbreviations

ABASI: Alexian Brothers Assessment of Self-Injury
ASQ: Ask Suicide-Screening Questions
IOP: intensive outpatient program
LOS: length of stay
NSSI: nonsuicidal self-injury
PHQ-A: Patient Health Questionnaire modified for adolescents

## Data Availability

Owing to the quality improvement nature of this study, participants did not agree to their data being shared publicly, so supporting data are not available.
